# Impact of Adiposity on Sensory Motor Performance in Adults Residing in a Hilly Area in Himachal Pradesh: A Neurophysiological Insight

**DOI:** 10.7759/cureus.91690

**Published:** 2025-09-05

**Authors:** Aanandita Sharma, Simran Sekhon, Jasdeep S Sandhu

**Affiliations:** 1 Physiology, Maharishi Markandeshwar Medical College & Hospital, Solan, IND

**Keywords:** adiposity, body composition, cognitive function, obesity, reaction time

## Abstract

Background: Increased body mass index (BMI) is a major health concern worldwide. BMI is an indicator of adiposity. Adiposity has been linked to various health complications, including cardiovascular disease, metabolic syndrome, and cognitive dysfunction in adults. Sensory motor performance of an individual is seen to be affected by adiposity. Sensory motor processing is the duration between presentation of a stimulus till response by an individual. Reaction time is an important tool to measure sensory motor response. The impact of increased adiposity level on cognitive function remains less clear. Hence, in the present study, the relationship between adiposity and sensorimotor performance among healthy adults was assessed.

Methodology: Anthropometric measurement parameters, including height, weight, and BMI, were measured in 300 participants aged 20-50 years. The participants were divided into three groups, i.e., normal, overweight, and obese. Sensory-motor performance of the subjects was assessed using a reaction time apparatus (MEDICAID-RTM 608, Medicaid Systems, India).

Results: Data analysis and results revealed a significant positive correlation between adiposity measures and slower reaction time in obese individuals as compared to the subjects with normal BMI, indicating that increased adiposity is associated with cognitive decline.

Conclusions: These findings suggest that excess body fat may negatively affect cognitive function, possibly due to altered neural processing speed. Further research is needed to explore varying mechanisms and potential interventions.

## Introduction

Adiposity refers to the accumulation of excessive body fat, which is commonly assessed through body mass index. Increased BMI is linked to various neurological, metabolic, and inflammatory changes that could influence reaction time in individuals [1]. While obesity and excessive adiposity have been extensively studied in relation to chronic diseases such as diabetes and cardiovascular conditions, their effects on cognitive responses remain less explored [2]. Cognition includes brain-based abilities of an individual that play a crucial role in everyday life. Some studies suggest that increased adiposity can lead to cognitive slowing as a result of adiposity-related systemic inflammation, altered neurotransmitter function, and metabolic dysregulation [3]. Reaction time (RT) is a tool to measure cognitive function, reflecting the speed at which an individual processes and responds to a particular stimulus. Cognitive function is essential; it plays a crucial role in daily activities and performance, occupational efficiency, and overall cognitive health of a person [4]. Many factors influence reaction time (RT), including age, gender, genetics, physical activity, and metabolic health of a person [5]. In recent years, attention has been given to the potential effect of adiposity on cognitive performance. From a physiological perspective, excess fat deposition is associated with very low-grade but chronic inflammation, which can be marked by increased levels of inflammatory markers, potentially leading to cognitive slowing [6]. In addition, adiposity-related changes in cerebral blood flow and insulin resistance may add to reduced cognitive functions and slower sensorimotor response [7]. Another possible mechanism involves neuromuscular function, and it has been associated with decreased nerve conduction velocity with impaired motor unit recruitment [8]. Increased adiposity level can also lead to increased oxidative stress, further affecting neuronal integrity and synaptic plasticity, which are crucial for rapid cognitive performance [9].

Given the rising prevalence of obesity and overweight conditions globally, understanding the relationship between adiposity and cognitive function is of significant importance. This study aimed to examine whether adiposity is associated with slower neural processing and performance in healthy young adults, an age group in which cognitive function is expected to be normal. By investigating this relationship, we can gain insights into the potential early effects of excess adiposity on cognitive performance, which may have implications for long-term cognitive health and functional abilities. Therefore, this study aimed to assess the relationship between adiposity and sensorimotor function in healthy adults.

## Materials and methods

Study setting and data collection

This cross-sectional study was conducted in the Department of Physiology in Maharishi Markandeshwar Medical College & Hospital (MMMC&H), Solan, Himachal Pradesh, India, from January 2023 to May 2025. This study included 300 healthy adults (age: 20-50 years, both sexes) after ethical approval from the Institutional Ethics Committee of MMMC&H, Solan (MMMCH/IEC/23/781) by a random sampling method.

The inclusion criteria are age of 20-50 years, apparently healthy individuals without any chronic illness (diabetes, hypertension, or cardiovascular disease) and had no acute illness at the time of recruitment, and willingness to participate 

The exclusion criteria are a history of neurological disorder, use of any cognitive-altering medications, metabolic disease such as uncontrolled diabetes or thyroid disorders, and impaired hearing. 

Measurement of anthropometric parameters

They were classified as the normal, overweight, and obese groups based on their BMI. Anthropometric parameters (BMI, body fat percentage, and waist-hip ratio (WHR)) were recorded. The study group was divided into three groups on the basis of the BMI: Group 1 (normal weight: BMI 18.5-24.9 kg/m²), Group 2 (overweight: BMI 25.0-29.9 kg/m²), and Group 3 (obese: BMI ≥30.0 kg/m²) between the age group of 20-50 years. The subjects were recruited on a voluntary basis, the procedure was explained to the subjects, and written informed consent was obtained. Height and weight were measured using a stadiometer and a calibrated weighing scale. Body fat percentage was measured using bioelectrical impedance analysis. BMI was calculated as weight (kg) / height (m²), and the waist-hip ratio (WHR) was calculated [10]. 

Assessment of sensory motor performance

For the assessment of sensory motor performance, a reaction time assessment was done. Reaction time was measured using a reaction time apparatus (RTM-MEDICAID 608, Medicaid Systems, India). In this apparatus, two types of stimuli were presented to the participant, i.e., visual reaction time and auditory reaction time. In VRT, three different colors (red, green, and yellow) were presented to the subject, and for ART (auditory reaction time), sounds of three different frequencies (low, medium, and high) were presented randomly. For each type of stimulus, three readings were taken, and the fastest of all was taken as the final reading for that particular stimulus [11].

Statistical analysis

Data were analyzed using IBM SPSS Statistics for Windows, version 25.0 (released 2012, IBM Corp., Armonk, NY). Continuous variables were expressed as mean ± SD. Pearson’s correlation assessed associations between adiposity measures and ART. Multiple regression was used to adjust for confounders (age, sex, physical activity). A p-value of 0.05 or less was considered statistically significant.

## Results

The demographic and anthropometric details of the study participants, categorized into normal, overweight, and obese BMI groups, are presented in Table 1. Each group comprised 100 individuals, with a balanced male-to-female distribution: 54/46 in the normal group, 56/44 in the overweight group, and 55/45 in the obese group. The mean age showed a slight upward trend from the normal group (34.2 ± 7.5 years) to the overweight (35.1 ± 6.9 years) and obese groups (35.8 ± 7.2 years), although the differences were minimal. 

**Table 1:** Demographic and anthropometric categories by BMI category *Data are presented as N, %, mean ± SD; BMI: body mass index; WHR: waist-hip ratio

Variable	Normal (n = 100)	Overweight (n = 100)	Obese (n = 100)
Age (years)	34.2 ± 7.5	35.1 ± 6.9	35.8 ± 7.2
Sex (M/F)	54/46	56/44	55/45
BMI (kg/m²)	21.7 ± 1.9	27.3 ± 2.3	32.8 ± 2.5
Body fat %	21.2 ± 3.6	28.9 ± 3.7	34.7 ± 4.2
WHR	0.82 ± 0.05	0.86 ± 0.02	0.96 ± 0.05

The BMI values demonstrated a clear and statistically notable gradient, increasing from 21.7 ± 1.9 kg/m² in the normal group to 27.3 ± 2.3 kg/m² in the overweight group and peaking at 32.8 ± 2.5 kg/m² in the obese group (Table 1). This was accompanied by a progressive rise in body fat percentage, with means of 21.2 ± 3.6%, 28.9 ± 3.7%, and 34.7 ± 4.2% for the normal, overweight, and obese categories, respectively. Similarly, the waist-hip ratio (WHR) increased from 0.82 ± 0.05 in the normal group to 0.86 ± 0.02 in the overweight group and 0.96 ± 0.05 in the obese group, suggesting greater central adiposity with increasing BMI classification.

Comparison of anthropometric variables and auditory reaction time

**Figure 1:**
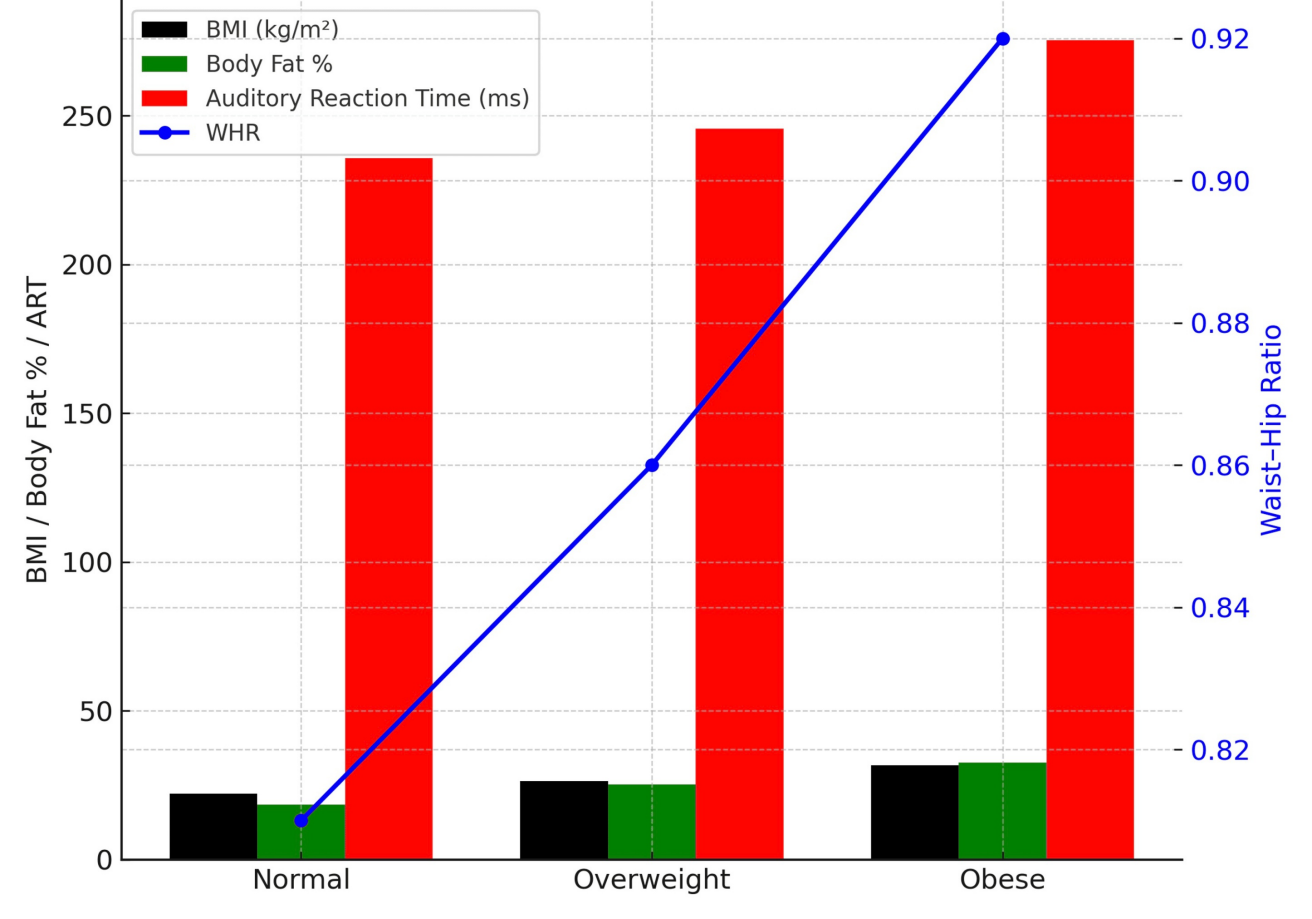
Bar graph showing the comparison of the BMI, body fat percentage, waist-hip ratio, and auditory reaction time across the BMI categories *Data are presented as Mean ± SD; ART: auditory reaction time; BMI: body mass index; WHR: waist-hip ratio

The visual comparison of BMI, body fat percentage, WHR, and auditory reaction time (ART) across BMI categories is depicted in Figure 1. The bar graph clearly demonstrates that individuals in the obese category exhibited the highest mean BMI, body fat percentage, WHR, and ART values. Overweight participants showed intermediate values for these parameters, while normal-weight individuals recorded the lowest values across all measures.

BMI and body fat percentage followed parallel trends, reflecting the direct relationship between these indices of adiposity. WHR displayed a notable upward shift from normal to overweight categories but rose more sharply in the obese group, indicating a disproportionate accumulation of abdominal fat in higher BMI categories. Importantly, ART showed a distinct increase from normal to obese groups, suggesting that higher adiposity is associated with slower sensory-motor responses.

To determine the independent predictors of auditory reaction time, a multiple regression analysis was performed with WHR, BMI, body fat percentage, and age as covariates (Table 2). WHR (β = 0.312, p = 0.001) and BMI (β = 0.228, p = 0.003) were significant positive predictors of auditory reaction time, while body fat percentage was a significant negative predictor (β = -0.187, p = 0.005). Age did not show a statistically significant association (p = 0.154). The model explained 38% of the variance in auditory reaction time (adjusted R² = 0.38, p < 0.001).

**Table 2:** Multiple regression analysis showing predictors of auditory reaction time in study participants. Multiple linear regression analysis showing the association of waist-hip ratio (WHR), body mass index (BMI), body fat percentage, and age with auditory reaction time in adults. β coefficient indicates the change in auditory reaction time per unit increase in the predictor variable. Significant predictors include WHR, BMI, and body fat percentage (p < 0.05).

Predictor variable	β coefficient	Standard error	95% confidence interval	p-value
WHR	0.312	0.085	0.145–0.479	0.001
BMI	0.228	0.073	0.084–0.372	0.003
Body fat %	-0.187	0.065	-0.315–-0.059	0.005
Age	0.041	0.029	-0.016–0.098	0.154

Detailed statistical comparisons and correlation results are presented in Table 3. ART values were lowest in the normal group (245.3 ± 28.8 ms), slightly higher in the overweight group (245.6 ± 32.5 ms), and highest in the obese group (284.6 ± 37.2 ms). The difference between the obese and normal groups was particularly marked, reflecting a considerable delay in reaction time among individuals with higher adiposity.

**Table 3:** Comparison of anthropometric parameters and auditory reaction time across bmi categories and their correlation with reaction time *Data are presented here as mean ± SD. p-values indicate significance of differences between groups.

Variable	Normal	Overweight	Obese	Correlation with reaction time (r)	p-value
BMI (kg/m^2^)	21.7± 1.9	27.3± 2.3	32.8 ± 2.5	0.31	0.011
Body fat percentage	21.2 ± 3.6	28.9 ± 3.7	34.7 ± 4.2	0.46	<0.001
WHR	0.82± 0.05	0.86 ± 0.02	0.96 ± 0.05	0.39	0.002
Auditory reaction time (ms)	245.3± 28.8	245.6 ± 32.5	284.6±37.2	—	—

The correlation analysis revealed significant positive relationships between ART and key anthropometric measures. BMI demonstrated a moderate positive correlation with ART (r = 0.31, p = 0.011), indicating that individuals with higher BMI tended to have slower reaction times. Body fat percentage showed a stronger correlation with ART (r = 0.46, p < 0.001), suggesting that fat mass might have a more pronounced impact on sensory-motor performance than BMI alone. WHR also exhibited a moderate positive correlation with ART (r = 0.39, p = 0.002), implying that central adiposity is an important determinant of delayed reaction time.

Trend observations

The data demonstrate a consistent upward trajectory in BMI, body fat percentage, WHR, and ART from the normal to obese categories. While BMI and body fat percentage increased steadily across categories, WHR showed a marked jump in the obese group, possibly indicating greater visceral fat accumulation. The increase in ART was not only statistically significant but also clinically relevant, given that delayed reaction times can impair daily functional performance and increase accident risk.

The relatively stronger correlation between ART and body fat percentage, compared to BMI, suggests that the distribution and composition of body mass - rather than body weight alone - may be more critical in influencing sensory-motor response times. Central adiposity, reflected in WHR, also emerged as an important predictor, potentially due to its metabolic and neurological implications.

## Discussion

The results of our study indicate that adiposity is linked with slower sensory-motor performance in adults. In this study, the sensory-motor performance of the subjects was assessed using a reaction time apparatus (MEDICAID RTM-608). Our findings indicate a significant relationship between adiposity and delayed sensory-motor performance, supporting the hypothesis that increased body fat negatively affects cognitive performance. These results are consistent with a previous study showing that individuals with higher body fat percentage exhibit slower sensorimotor response [12]. Similar results have been shown by other studies, showing that VRT (visual reaction time) was prolonged in the overweight or the obese individuals as compared to the subjects with normal BMI, and the result was statistically significant [13,14]. An increase in adiposity has been linked to a change in cognitive performance in adults, including executive function and processing speed. This change in cognitive function may be accelerated by various mechanisms such as insulin resistance and systemic inflammation, along with some changes in cerebrovascular functions [15]. It has also been reported by previous studies that adiposity-related inflammatory markers, such as IL-6 and CRP (C-reactive protein), can negatively impact neuronal processing. Moreover, it is reported that BDNF is associated with increased adiposity, which has a key role in synaptic plasticity and cognitive function [16,17]. Slowing of reaction time could be due to the fact that excess adiposity may impair nerve conduction velocity due to systemic inflammation increase and metabolic dysregulation [18]. In addition to obesity-related alterations in neurotransmitter systems, acetylcholine and dopamine may be contributing factors to slower reaction time and impaired cognitive function [19]. Potential mechanisms include systemic inflammation, altered neurotransmitter function, and reduced cerebral blood flow [20,21]. It has been documented that an increased level of IL-6 is a potential marker for insulin resistance, which can alter cerebral glucose metabolism; as a result, it could lead to cognitive decline. In addition, reduced cerebral perfusion and increased oxidative stress in adiposity can disrupt neural connectivity. The results of our study suggest that sensory motor performance is slowed by increased adiposity, and hence, adiposity is negatively associated with cognitive function.

Furthermore, recent research has highlighted that the impact of adiposity on cognitive and sensory-motor functions is not solely mediated through metabolic and inflammatory pathways, but also via vascular dysfunction. Obesity is known to cause endothelial damage, reduce nitric oxide bioavailability, and promote arterial stiffness, all of which can impair cerebral blood flow regulation [22]. Reduced cerebral perfusion has been associated with slower neural processing speed and delayed reaction times, consistent with our findings. In this context, adiposity-related vascular dysfunction may exacerbate the already heightened risk of cognitive decline in obese individuals.

In addition, mitochondrial dysfunction has emerged as a critical link between increased adiposity and impaired brain function. Mitochondria are essential for ATP generation in neurons, and their dysfunction can compromise synaptic transmission and plasticity [23]. Excess fat accumulation increases oxidative stress, leading to mitochondrial DNA damage and impaired neuronal energy metabolism. These cellular changes may contribute to the prolonged auditory reaction time observed in our obese participants, as efficient energy metabolism is fundamental for rapid neural signaling.

Another potential pathway involves altered hormonal signaling. Adipose tissue acts as an endocrine organ, secreting adipokines such as leptin, adiponectin, and resistin, which influence brain function [24]. While leptin has neuroprotective effects under physiological conditions, chronic hyperleptinemia in obesity can lead to leptin resistance, reducing its beneficial effects on synaptic plasticity and cognitive performance. Similarly, decreased adiponectin levels, commonly found in obesity, are associated with increased inflammation and insulin resistance, both of which are detrimental to brain health.

Neuroinflammation plays a pivotal role in mediating the effects of adiposity on the nervous system. Microglial activation in response to obesity-induced systemic inflammation has been shown to impair synaptic plasticity and increase neurodegeneration risk [25]. Chronic activation of microglia leads to sustained production of pro-inflammatory cytokines such as IL-1β, TNF-α, and IL-6, which interfere with long-term potentiation - a key mechanism for learning and memory. This neuroinflammatory environment could explain the observed delay in reaction times among overweight and obese individuals in our study.

Beyond biological mechanisms, lifestyle factors commonly associated with obesity may also play a role. Reduced physical activity, poor sleep quality, and suboptimal diet patterns - frequently reported among individuals with higher adiposity - can each independently impair cognitive performance [26]. For instance, diets high in saturated fats and refined sugars are linked to hippocampal dysfunction and reduced memory performance. Sleep disturbances, including obstructive sleep apnea, are prevalent in obese populations and can cause intermittent hypoxia, leading to white matter damage and slowed neural transmission [20].

It is also important to consider the bidirectional relationship between adiposity and cognitive function. While obesity can lead to slower reaction times and reduced executive function, impaired cognitive control may in turn contribute to unhealthy lifestyle choices, creating a self-perpetuating cycle [27]. This emphasizes the need for early intervention strategies targeting both weight management and cognitive health.

Interestingly, sex differences may influence the relationship between adiposity and cognitive outcomes. Some studies have reported that women with higher body fat percentages exhibit more pronounced declines in processing speed compared to men, possibly due to differences in fat distribution, hormonal fluctuations, and susceptibility to neuroinflammation [28]. Although our study had a nearly equal male-to-female ratio across groups, future analyses could investigate whether sex-specific patterns exist in our dataset.

In summary, our extended analysis supports the growing body of evidence that adiposity exerts multifactorial negative effects on cognitive and sensory-motor performance. Mechanisms likely involve vascular dysfunction, mitochondrial impairment, altered hormonal signaling, neuroinflammation, lifestyle factors, and central adiposity. The strong correlations between body fat percentage, WHR, and auditory reaction time observed in our study reinforce the need for preventive and therapeutic strategies that address both obesity and cognitive health. Future research should explore longitudinal outcomes and intervention efficacy to better inform clinical practice.

Clinical implication

These findings suggest that obesity is a modifiable risk factor for cognitive decline. Incorporating weight management strategies may help preserve cognitive health and daily functional performance. 

Limitations

The study sample may not represent the wider population. Inflammatory markers and neuroimaging techniques were not included, limiting mechanistic insights. Future studies should incorporate MRI and biomarker profiling and examine sex-specific effects.

## Conclusions

This study demonstrates that increased adiposity is associated with slower reaction time in healthy adults, indicating an inverse relationship between adiposity and sensory-motor performance and neural processing. Given the importance of cognitive function in daily activities, strategies to reduce adiposity could be beneficial for maintaining cognitive performance. Increased adiposity is significantly associated with slower auditory reaction time in adults, indicating reduced neural processing speed. Public health strategies targeting adiposity reduction could help maintain cognitive performance.
